# T_1ρ_ for Radiotherapy Treatment Response Monitoring in Rectal Cancer Patients: A Pilot Study

**DOI:** 10.3390/jcm11071998

**Published:** 2022-04-02

**Authors:** Ernst S. Kooreman, Max Tanaka, Leon C. ter Beek, Femke P. Peters, Corrie A. M. Marijnen, Uulke A. van der Heide, Petra J. van Houdt

**Affiliations:** 1 Department of Radiation Oncology, The Netherlands Cancer Institute, 1066 CX Amsterdam, The Netherlands; e.kooreman@nki.nl (E.S.K.); m.tanaka@nki.nl (M.T.); f.peters@nki.nl (F.P.P.); c.marijnen@nki.nl (C.A.M.M.); u.vd.heide@nki.nl (U.A.v.d.H.); 2 Department of Radiology, The Netherlands Cancer Institute, 1066 CX Amsterdam, The Netherlands; l.t.beek@nki.nl

**Keywords:** T1rho, quantitative MRI, treatment response monitoring, MR-linac

## Abstract

Quantitative MRI has the potential to produce imaging biomarkers for the prediction of early response to radiotherapy treatment. In this pilot study, a potential imaging biomarker, the T_1ρ_ relaxation time, is assessed for this purpose. A T_1ρ_ sequence was implemented on a 1.5 T MR-linac system, a system that combines an MRI with a linear accelerator for radiation treatment. An agar phantom with concentrations of 1–4% *w*/*w* was constructed for technical validation of the sequence. Phantom images were assessed in terms of short-term repeatability and signal-to-noise ratio. Twelve rectal cancer patients, who were treated with 5 × 5 Gy, were imaged on each treatment fraction. Individual changes in the T_1ρ_ values of the gross tumor volume (GTV) showed an increase for most patients, although a paired *t*-test comparing values in the GTV from the first to the last treatment fraction showed no statistically significant difference. The phantom measurements showed excellent short-term repeatability (0.5–1.5 ms), and phantom T_1ρ_ values corresponded to the literature values. T_1ρ_ imaging was implemented successfully on the MR-linac, with a repeatability comparable to diagnostic systems, although clinical benefit in terms of treatment response monitoring remains to be demonstrated.

## 1. Introduction

The conventional assessment of treatment response to radiation therapy involves re-evaluating the tumor using MRI or CT, and is largely based on morphological change. However, changes in the tumor microenvironment, such as changes in protein concentration, already happen directly after irradiation, over a much shorter time scale than morphological changes [1]. This tumor microenvironment may be imaged using quantitative MRI (qMRI), and thus, early changes in qMRI metrics could potentially be used as quantitative imaging biomarkers (QIBs) for early treatment response assessment [2]. With the introduction of MR-linacs—hybrid machines that combine an MRI with a linear accelerator for radiation treatment—the acquisition of qMRI at each treatment fraction is possible. This creates a platform wherein potential novel qMRI biomarkers can be searched for and evaluated with a limited increase in patient burden.

One such potential biomarker is called T_1ρ_, which stands for T_1_ relaxation in the rotating frame. T_1ρ_ is the relaxation time of spins while under the influence of a continuous RF pulse, which is mainly influenced by protein–water interactions and, therefore, sensitive to the presence of protein molecules in tissue [3,4,5]. Some preliminary studies have shown the potential of T_1ρ_ as a QIB for the treatment response monitoring of different kinds of cancer treatment. In a preclinical study, Hectors et al. demonstrated changes only 3 days after treatment, as a result of high-intensity focused ultrasound treatment in mice with murine colon carcinoma [6]. In humans, T_1ρ_ was demonstrated to be able to distinguish between a tumor and the peripheral zone in prostate cancer [7], and between tumor, fat, and fibrosis in freshly excised breast tissue [8]. In terms of treatment response monitoring, an increase in T_1ρ_ was found in healthy parotids during radiotherapy treatment of nasopharyngeal cancer patients [9].

The aim of the current study is to explore T_1ρ_ as a potential QIB for treatment response monitoring. In order to achieve this, a phantom was constructed and measured for technical validation purposes, and a T_1ρ_ sequence was scanned in rectal cancer patients to show clinical feasibility.

## 2. Materials and Methods

### 2.1. MRI Sequence

In this study, the Unity MR-linac (Elekta AB, Stockholm, Sweden) was used. This system integrates a 1.5 T MRI with a linear accelerator. The MRI system is based on a Philips Ingenia system (Philips Healthcare, Best, The Netherlands), with adaptations made to allow for patient irradiation [10]. The gradient coils of the MRI are physically split to allow the radiation to pass through, and the system uses an 8-channel radio-translucent phased-array receive coil [11].

With T_1ρ_, after excitation, a continuous RF pulse (the spin-lock pulse) is applied on-resonance along the magnetization vector in the transverse plane. This spin-lock pulse is a weak magnetic field (in the μT range) that rotates with the spins at the Larmor frequency. The spins then relax towards a new equilibrium state associated with the spin-lock pulse, with the T_1ρ_ relaxation time constant.

A T_1ρ_ sequence consisting of a spin-lock pulse cluster followed by a TSE readout was implemented. The ΔB_0_ and B_1_ insensitive spin-lock cluster, as described by Witschey et al., was used [12], with a spin-lock amplitude of 400 Hz. Six images were acquired with spin-lock times (TSL) of 0, 5, 10, 20, 40, and 60 ms. Due to software limitations, short gaps of 0.6 ms had to be introduced after the excitation and around the refocusing pulses, and spin-lock pulses exceeding 10 ms were interrupted with these gaps every 10 ms. The T_1ρ_ pre-pulse was followed by a crusher gradient, and a single-shot TSE sequence was used for readout. The FOV was 420 × 420 × 104 mm^3^, with acquisition voxel sizes of 3 × 3 × 5 mm^3^. Partial Fourier was used with a factor of 0.6, resulting in an echo train length of 84. The number of acquired slices was 19 with a gap of 0.5 mm between each slice. TR/TE were 3000/4.2 ms and the scan time was 57 s per spin-lock time. The total scan time for the complete T_1ρ_ scan was 5 min and 42 s. To calculate a T_1ρ_ map, a straight line was fitted to the logarithm of the signal intensity values using weighted least squares on a voxel-by-voxel basis.

### 2.2. Phantom

**Figure 1:**
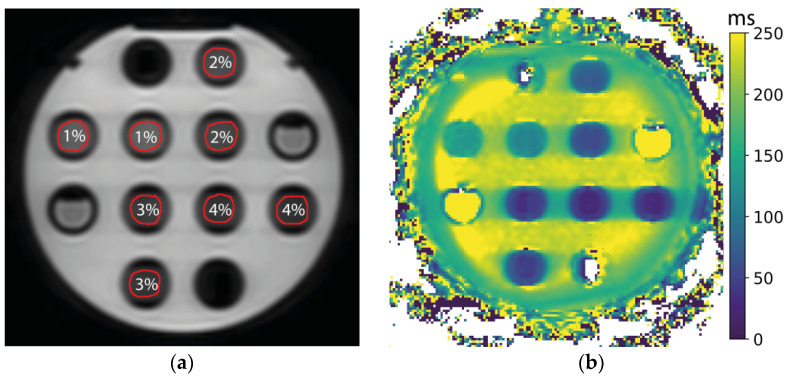
Example phantom measurement: (**a**) The phantom is shown as measured with a spin-lock time of 0 ms, and the percentages indicate the agar concentration in the tubes. The unmarked tubes contain distilled water; (**b**) T_1ρ_ map of the phantom shown in (**a**).

An agar phantom was created in a similar fashion to Buck et al. [13]. Agar powder was diluted in hot, distilled water to create agarose gel stock solutions with concentrations of 1, 2, 3, and 4% *w*/*w*. The mixtures were poured into 30 mL tubes when still warm. Two tubes were created from the same stock solution for each concentration. These tubes were placed in a custom-made holder filled with a copper-sulfate solution. Figure 1 shows the placement of the tubes. The phantom was scanned at room temperature, while placed in the iso-center of the bore.

As, to date, there are no guidelines for T_1ρ_ validation with phantoms, we followed the phantom validation framework as described in the diffusion profile of the Quantitative Imaging Biomarkers Alliance (QIBA) [14]. The agar phantom was scanned four times, consecutively, in one scan session. This allowed for the determination of short-term phantom repeatability and the signal-to-noise ratio (SNR), based on the variation in signal intensities between the four consecutive scans. For the analysis, a region of interest (ROI) was delineated for each tube on the center slice. As there is no gold standard for measuring T_1ρ_, the accuracy of the sequence could not be determined. The repeatability coefficient (RC) was determined as $\mathrm{RC}=2.77\sigma_{w}$ and the within-subject (phantom tube) coefficient of variation (wCV) as $\mathrm{wCV}=100\%\sigma_{w}/\mu$. Here, $\sigma_{w}$ is the within-subject standard deviation and μ the mean, both calculated from the mean values of the tube ROIs of the repeated measurements. 

For the SNR of each tube, voxel-wise standard deviation (SD) maps and mean maps were calculated from the repeated measurements to make a temporal noise map and a temporal mean map. The SNR was then calculated for each tube by dividing the ROI means of the temporal mean map by the ROI means of the temporal noise map. The 95% confidence intervals for these SNR estimates were calculated as $\pm 1.96\sigma_{\mathrm{SNR}}/\sqrt{N}$, where N is the number of voxels in the ROI, and $\sigma_{\mathrm{SNR}}=\mathrm{SNR}\sqrt{{\mathrm{mCV}}^{2}+{\mathrm{nCV}}^{2}}$. Here SNR is the SNR of the tube, and mCV and nCV are the coefficients of variation (SD/mean) of each ROI in the temporal mean map and temporal noise map, respectively [14].

To see if there are differences between tubes with the same agar concentration but a different location in the phantom, a two-sided *t*-test was performed per agar concentration on the voxel values inside the ROIs from the first acquisition.

### 2.3. Patients

Twelve intermediate-risk rectal cancer patients who received 5 × 5 Gy external beam radiation therapy over one week were included in this study. The target volumes included the mesorectum and elective lymph nodes. All patients were treated on a Unity MR-linac, allowing them to be imaged at each treatment fraction. The study was approved by the local ethics committee and all patients gave written informed consent.

A 3D-TSE T_2_-weighted anatomical scan was acquired right before the T_1ρ_ scan for delineation purposes. Scan parameters included TR/TE = 1300/123 ms, a FOV of 400 × 449 × 250 mm^3^, and acquisition voxel sizes of 1.8 × 1.8 × 1.8 mm^3^. A SENSE factor of 4 was used, as well as partial Fourier with a factor of 0.6 in one direction, and 0.7 in a second direction, for a scan time of 1 min 58 s.

A patient follow-up was available for 10 to 15 months after treatment. Patients were classified as complete responders or incomplete responders. Complete responders were either patients with pathological a complete response after surgery (ypT0N0), or patients with a sustained clinical complete response (cCR) during the available follow up time. cCR was defined as no or minimal residual tumor after neoadjuvant therapy, based on digital rectal examination, endoscopy, and MRI [15]. Patients with ypT1-T4 after surgery were classified as incomplete responders. Patient characteristics are given in Table 1.

For all patients, the gross tumor volume (GTV), as visible on MRI, was delineated at each treatment fraction. As a control, a region of the mesorectum close to the tumor was delineated, as were both femoral heads. These delineations were propagated to the T_1ρ_ maps, and median values from the delineations were used for further analysis. The RC was calculated in the femoral heads, using the $\sigma_{w}$ from all treatment fractions.

To test for differences in the T_1ρ_ values of the GTV between complete- and incomplete-responders, a *t*-test was used on the data from the first fraction, and separately on the data from the last fraction. To test for differences between values from all ROIs between the first and last fractions, a paired *t*-test was used.

All statistical analyses were performed in R (v3.6.1) with the mixed effects model implementation of the lme4 package [16]. Statistical significance was assumed when α < 0.05.

## 3. Results

### 3.1. Phantom Measurements

An image of the T_1ρ_ map of the first phantom measurement is shown in Figure 1. 

Mean values from the ROIs of the center slice, the repeatability measures, and the SNR are presented in Table 2. The average RC was 0.9 ms, and the average wCV was 0.5%. All the outer tubes, positioned further away from the iso-center, show consistently higher T_1ρ_ values than their inner counterparts. Two-sided *t*-tests show that these differences are significant (all *p* < 0.001).

### 3.2. Patients

**Figure 2:**
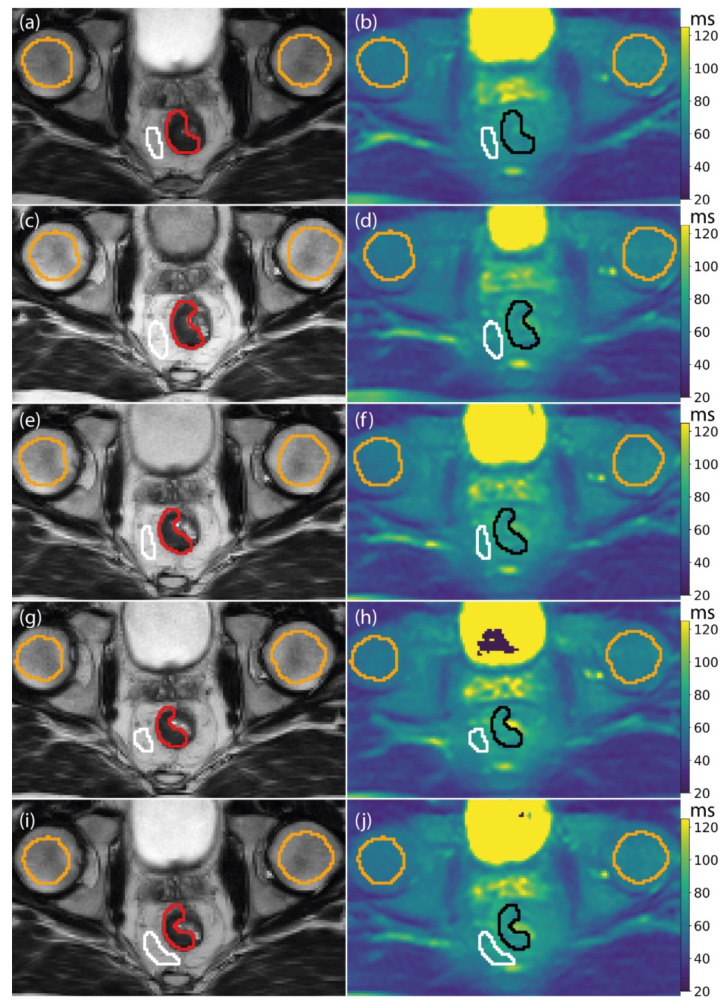
Example T_1ρ_ maps of a single patient. Each row corresponds to a different treatment fraction. On the left, (**a**,**c**,**e**,**g**,**i**) the T_2_-weighted image used for delineation is shown, with the corresponding T_1ρ_ maps on the right (**b**,**d**,**f**,**h**,**j**). The gross tumor volume (GTV) is shown in red on the T_2_-weighted image and in black on the T_1ρ_ image. The mesorectum region of interest (ROI) is shown in white, and the femoral heads are shown in orange. The T_1ρ_ maps are shown on a color scale from 20 to 120 ms.

All 12 patients were successfully scanned at each treatment fraction for a total of 60 fractions. An example T_1ρ_ map from all the fractions of a single patient is shown in Figure 2. 

Baseline T_1ρ_ values, measured on the first fraction before receiving the first radiation dose, were 77 ± 8 ms (mean ± SD of all patients) for the GTV; 73 ± 11 ms for the mesorectum; 64 ± 4 ms for the left femoral head; and 59 ± 5 ms for the right femoral head. The RC in both femoral heads was 4 ms.

**Figure 3:**
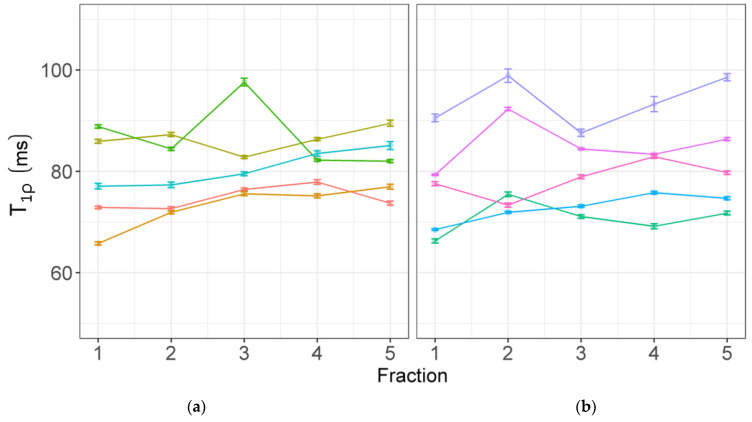
T_1ρ_ relaxation times from individual patients for each fraction; (**a**) complete responders; (**b**) incomplete responders. The mean ± standard error of the mean is shown from all voxels inside the GTV. Each line (and color) shows a different patient.

Figure 3 shows the change in T_1ρ_ values during treatment from the GTVs of the complete- (a) and incomplete–responders (b). The median number of voxels in these GTVs is 567 (range: 122–3215). Most patients show an increasing trend in T_1ρ_ values. In addition, individual histograms of the voxels from the GTVs of all patients on all treatment fractions can be found in Supplemental Figure S1. 

Table 3 contains the averaged values over all patients for each ROI at each treatment fraction. The GTV is additionally split into complete- and incomplete-responders. Values in the femoral heads are relatively stable over the course of treatment. There is a difference between the left and right femoral heads, where the T_1ρ_ in the left femoral head is consistently higher. In the GTV, a slight increase in the average can be seen, and the T_1ρ_ relaxation time in the GTV is consistently higher than in the nearby mesorectum. The change in T_1ρ_ values between the first and the last treatment fraction is given in the last column, and paired *t*-tests show a statistically significant difference for the GTV values of the incomplete responders (*p* = 0.02). A *t*-test for the difference in the T_1ρ_ values from the GTV between the complete and incomplete responders at the first treatment fraction showed no statistically significant difference.

## 4. Discussion

In this pilot study, we investigated T_1ρ_ as a potential QIB for treatment response monitoring. A ΔB_0_ and B_1_ insensitive spin-lock pulse was adapted slightly for use on the Unity MR-linac system. With the agar phantom measurements, it was shown that T_1ρ_ relaxation times can be quantified on the MR-linac, and the feasibility of acquiring a T_1ρ_ map at each treatment fraction was demonstrated in twelve rectal cancer patients.

The first step in technical validation is to assess the accuracy and repeatability of the qMRI measurements in phantoms. The T_1ρ_ sequence was adapted by inserting short interruptions in the normally continuous spin-lock pulse. It is conceivable that interruptions influence the T_1ρ_ relaxation time, or cause a loss of spin-lock. In this sense, it is encouraging that the T_1ρ_ values of our phantom measurements decrease with an increasing agar concentration, as shown in previous studies. In their study, Buck et al. found a decrease from 55 to 29 ms in an agar phantom for concentrations of 2–4% [13], compared to 59 to 28 ms for the same concentrations in the inner tubes in this study.

To assess whether such a new technique has potential for treatment response measurements, the next step is to determine whether changes occur during treatment. In this study, some increase in the T_1ρ_ values can be observed in the GTVs of individual patients, although the change between the first and last treatment fraction on a group level was not statistically significant. Although a statistically significant change was found between the T_1ρ_ values from the GTVs of incomplete responders from the first fraction versus the last fraction, this should be carefully interpreted, as multiple tests were performed and the number of patients in this group was low. More patients would need to be assessed to determine if the increase in T_1ρ_ indeed holds.

Other uses of T_1ρ_ are possible. For instance, T_1ρ_ has been shown to be valuable for fibrosis detection in the liver, parotid glands, and breast [8,9,17]. Fibrosis is also an important factor in the response evaluation of rectal cancer, and in particular, differentiating between tumor and fibrosis remains difficult [18]. In this regard, it might be interesting to explore T_1ρ_ voxel maps. 

The T_1ρ_ relaxation time depends on the strength of the spin-lock pulse, and by using different strengths, the T_1ρ_ relaxation time reflects different parts of the biological microenvironment. It is probable that the 400 Hz used in this study is not optimal for treatment response purposes in rectal cancer patients.

The RC from femoral heads corresponds to previously reported values, albeit in other tissues. In the head and neck, an RC of 2.3–5.2 ms was found for different organs [19], and a study of the prostate reported values between 13 and 23% relative to the median value of different prostate regions (which would be 7% for both femoral heads in this study) [7]. Values from cartilage studies reported RCs of 0–18% [19]. The RCs reported here indicate that T_1ρ_ measurements can be performed as reliable on the Unity MR-linac as on diagnostic MRI systems.

A consistent difference between the left and the right femoral heads was found, and also between the inner and outer tubes containing the same solution of agar in the phantom. This indicates that there might be an influence of the spatial location inside the MR-linac on the T_1ρ_ measurement, similar to diffusion MRI [20]. This could be related to magnetic field inhomogeneities and would be worth investigating in the future.

In conclusion, in this pilot study, a T_1ρ_ sequence was implemented and evaluated on an MR-linac system. The phantom measurements showed high repeatability and T_1ρ_ values corresponded to those in the literature, although spatial variation was present. Additionally, T_1ρ_ maps of rectal cancer patients were successfully acquired at each treatment fraction, with a repeatability comparable to diagnostic systems. Validation in a larger cohort is desired to establish if T_1ρ_ could be of clinical benefit in terms of treatment response monitoring.

## Figures and Tables

**Table 1:** Patient age and T-stage, sorted by response status. Age is presented as median (range).

Complete Response (*n* = 5)	
Age	61 (52–72)
T-stage	
T2N0	2
T3aN0	1
T3aN2	1
T3bN1	1
**Incomplete Response (*n* = 5)**	
Age	53 (34–64)
T-stage	
T2N0	2
T3bN0	1
T3cN1	1
T4bN1	1
**Response Unknown (*n* = 2)**	
Age	61, 73
T-stage	
T3bN0	1
T3bN1	1

**Table 2:** Metrics derived from the phantom measurements: Inside indicates a tube positioned in the inner four spaces of the phantom holder and outside indicates tubes placed close to the edge of the phantom. The mean and SD were calculated from the voxels inside the ROIs in the first scan. RC = repeatability coefficient, wCV = within-tube coefficient of variation, SNR = signal-to-noise ratio.

Tube	Mean ± SD (ms)	RC (ms)	wCV (%)	SNR ± 95% CI
1% inside	113 ± 2	0.5	0.1	96 ± 11
1% outside	136 ± 5	1.5	0.4	73 ± 9
2% inside	59 ± 3	0.6	0.4	83 ± 10
2% outside	76 ± 6	1.3	0.6	101 ± 11
3% inside	39 ± 5	0.8	0.7	37 ± 4
3% outside	48 ± 6	1.4	1.1	45 ± 6
4% inside	28 ± 4	0.7	0.9	36 ± 7
4% outside	32 ± 5	0.8	0.9	36 ± 5

**Table 3:** Group mean T_1ρ_ values for each ROI and treatment fraction: The values are presented in ms as mean ± standard error of the mean (SEM). The last column shows the mean ± SEM of the paired differences between fraction 1 and fraction 5. The *p*-values for the difference between the first and last fraction were calculated using a paired *t*-test.

ROI	Fraction 1	Fraction 2	Fraction 3	Fraction 4	Fraction 5	Fraction 5−Fraction 1
GTV						
All (*n* = 12)	77 ± 2	79 ± 2	80 ± 2	80 ± 2	81 ± 2	4 ± 1 (*p* = 0.13)
Complete responders (*n* = 5)	77 ± 4	77 ± 3	81 ± 3	80 ± 2	80 ± 2	3 ± 3 (*p* = 0.44)
Incomplete responders (*n* = 5)	76 ± 4	79 ± 4	78 ± 3	80 ± 4	81 ± 5	5 ± 1 (*p* = 0.02)
Mesorectum	73 ± 3	72 ± 3	75 ± 3	76 ± 3	75 ± 3	2 ± 1 (*p* = 0.24)
Femoral head left	64 ± 1	65 ± 2	65 ± 1	66 ± 2	65 ± 1	1 ± 1 (*p* = 0.46)
Femoral head right	59 ± 1	59 ± 1	59 ± 1	60 ± 2	59 ± 2	1 ± 1 (*p* = 0.10)

## Data Availability

The data presented in this study are available on request from the corresponding author.

## Supplementary Materials

The following supporting information can be downloaded at: https://www.mdpi.com/article/10.3390/jcm11071998/s1, Figure S1: Histograms of the GTVs of all 12 patients and all treatment fractions are shown.
