# Supplementary material for: T1ρ for Radiotherapy Treatment Response Monitoring in Rectal Cancer Patients: A Pilot Study
Source: J Clin Med. 2022 Apr 2;11(7):1998. doi: 10.3390/jcm11071998 (PMC8999631; doi:10.3390/jcm11071998)

Supplementary Material to "$T_{1\rho}$ for Radiotherapy Treatment Response Monitoring in Rectal Cancer Patients: A Pilot Study" by E.S. Kooreman *et al.*

Supplemental Figure S1: Histograms of the GTVs of all 12 patients and all treatment fractions are shown.

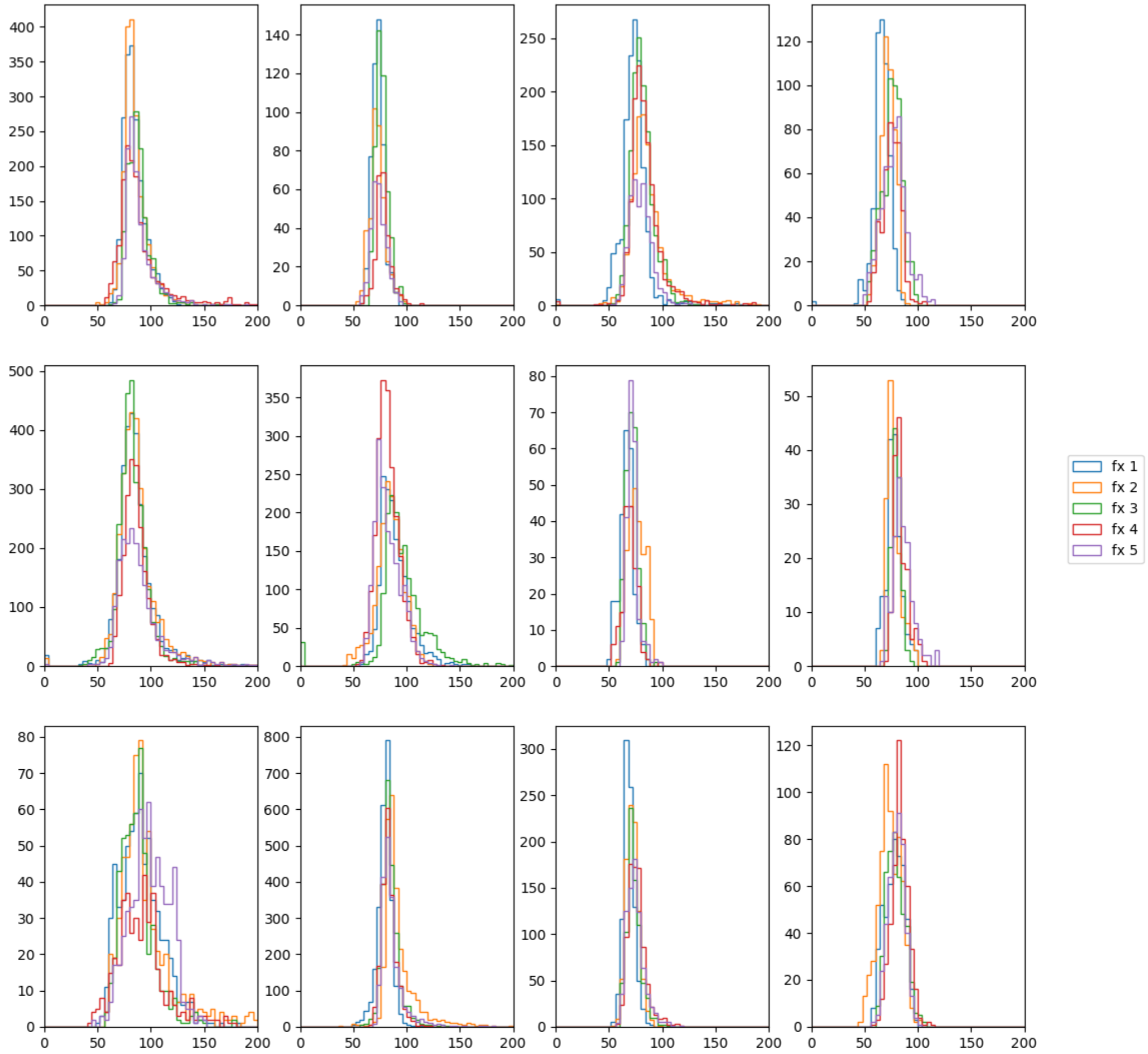

Supplement: Supplementary file 1 [file jcm-11-01998-s001.zip › jcm-1639202-supplementary.pdf]
